# Drought-Induced Amplification of *Saint Louis encephalitis virus*, Florida

**DOI:** 10.3201/eid0806.010417

**Published:** 2002-06

**Authors:** Jeffrey Shaman, Jonathan F. Day, Marc Stieglitz

**Affiliations:** * Columbia University, New York, New York, USA; † University of Florida, Vero Beach, Florida, USA

**Keywords:** Saint Louis encephalitis, amplification, transmission, entomology, Culex nigripalpus, hydrology, drought, dynamic modeling, forecast

## Abstract

We used a dynamic hydrology model to simulate water table depth (WTD) and quantify the relationship between *Saint Louis encephalitis* *virus* (SLEV) transmission and hydrologic conditions in Indian River County, Florida, from 1986 through 1991, a period with an SLEV epidemic. Virus transmission followed periods of modeled drought (specifically low WTDs 12 to 17 weeks before virus transmission, followed by a rising of the water table 1 to 2 weeks before virus transmission). Further evidence from collections of *Culex nigripalpus* (the major mosquito vector of SLEV in Florida) suggests that during extended spring droughts vector mosquitoes and nestling, juvenile, and adult wild birds congregate in selected refuges, facilitating epizootic amplification of SLEV. When the drought ends and habitat availability increases, the SLEV-infected *Cx. nigripalpus* and wild birds disperse, initiating an SLEV transmission cycle. These findings demonstrate a mechanism by which drought facilitates the amplification of SLEV and its subsequent transmission to humans.

Florida is vulnerable to epidemic transmission of *Saint Louis encephalitis virus* (SLEV). Five epidemics (>20 human cases each) of SLEV have been recorded in south Florida since 1952 (*1*). The most recent epidemic occurred in 1990 when 226 cases were reported throughout south-central Florida. The ability to accurately forecast SLEV epidemics is needed to minimize human health risks and focus vector control efforts. The development of such forecasting capabilities, however, requires complete understanding of the mosquito vector and amplification-host interactions that result in virus transmission to humans.

The annual SLEV transmission cycle in south Florida can be divided into four phases: January–March, maintenance; April–June, amplification; July–September, early transmission; and October–December, late transmission (*2*). The amplification phase involves the epizootic cycling of SLEV between mosquito vectors and avian amplification hosts. Amplification is necessary to achieve mosquito infection rates sufficient to cause human epidemics (*3*). In Florida, resident juvenile and nestling wild birds serve as the primary amplification host of SLEV (*4*). Nestling and juvenile birds are excellent amplification hosts because of their inefficient, poorly developed immune systems; their sparse feather coverage, which allows large numbers of mosquitoes to feed; and their lack of defensive behavior toward blood-feeding mosquitoes (*4*). Evidence also suggests that young birds may have elevated and extended viremias compared with their adult conspecifics (*3*), further facilitating SLEV amplification.

Others have proposed that SLEV epidemics may result from a specific combination of biotic and abiotic conditions that favor early season virus amplification followed by transmission (*1*). Several meteorologic variables have been associated with the amplification and transmission of SLEV and with vector abundance (*5*,*6*). High temperature accelerates the rate of pathogen and vector development, and high humidity increases vector flight and host-seeking behaviors (*6*).

Particular attention has been focused on precipitation, which is necessary for the formation of mosquito breeding habitats. In Florida, *Culex nigripalpus* Theobald is the epidemic and epizootic vector of SLEV (*7*–*9*). Provost (*10*) suggested that droughts during the *Cx. nigripalpus* breeding season, followed by heavy rainfall and high humidity, may favor SLEV transmission. More recent studies have shown that summer and autumn rainfall patterns are correlated with SLEV transmission (*11*), blood feeding (*12*), oviposition (*13*), and abundance (*2*). The association of rainfall with virus transmission provides a working model for the prediction of SLEV transmission to humans in Florida.

The availability of mosquito breeding habitats, however, can be more directly assessed by using current hydrologic modeling techniques to track temporal variations in water table depth (WTD). Such techniques have been used to predict mosquito abundance in temperate settings *(14)*. We expanded on this approach, applying these methods to predict both *Cx. nigripalpus* abundance and SLEV transmission dynamics in Florida.

For this study, we used a dynamic hydrology model (*15*) to simulate daily WTD in the Vero Beach area of Indian River County, Florida, which was the epicenter of the 1990 Florida SLEV epidemic (*16*). We then evaluated the association of WTD with SLEV transmission to sentinel chickens from 1986 to 1991. Modeled daily, WTD was also compared with field collections of *Cx. nigripalpus* taken in Indian River County during the same time period.

## Modeling Overview and Methods

Variations of WTD in space and time determine where and when pools of water form at the land surface, thus creating potential mosquito breeding habitats. WTD, however, is not merely a function of precipitation. Other meteorologic variables, as well as soil and vegetation type and antecedent conditions, must be considered if evapotranspiration, water movement within the soil column, and river runoff are to be quantified. Topography must also be constrained if the flow of water across the land surface, runoff rates, and the local convergence of water in lowlands (surface pooling) are to be modeled accurately.

We combined a soil column model, which simulates the vertical movement of water and heat within the soil and between the soil surface, plus vegetation and the atmosphere, with the TOPMODEL (TOPography-based hydrology MODEL) approach (*17*–*20*), which incorporates topographic data to track the horizontal movement of shallow groundwater from the uplands to the lowlands. TOPMODEL formulations permit dynamically consistent calculations of both the saturated fraction within the watershed (partial contributing area) and the groundwater flow that supports this area, from knowledge of the mean depth of the water table and a probability density function for soil moisture deficit derived from topographic statistics. Using the model, we can produce a three-dimensional picture of soil moisture distribution within a catchment. This approach to modeling the land surface has been validated at several catchments, ranging in scale from the Red Arkansas Basin (570,000 km^2^) (*21*) to the Black Rock Forest catchment (1.34 km^2^) (*22*).

## Data Collection and Analysis

### SLEV Transmission Data

**Figure 1:**
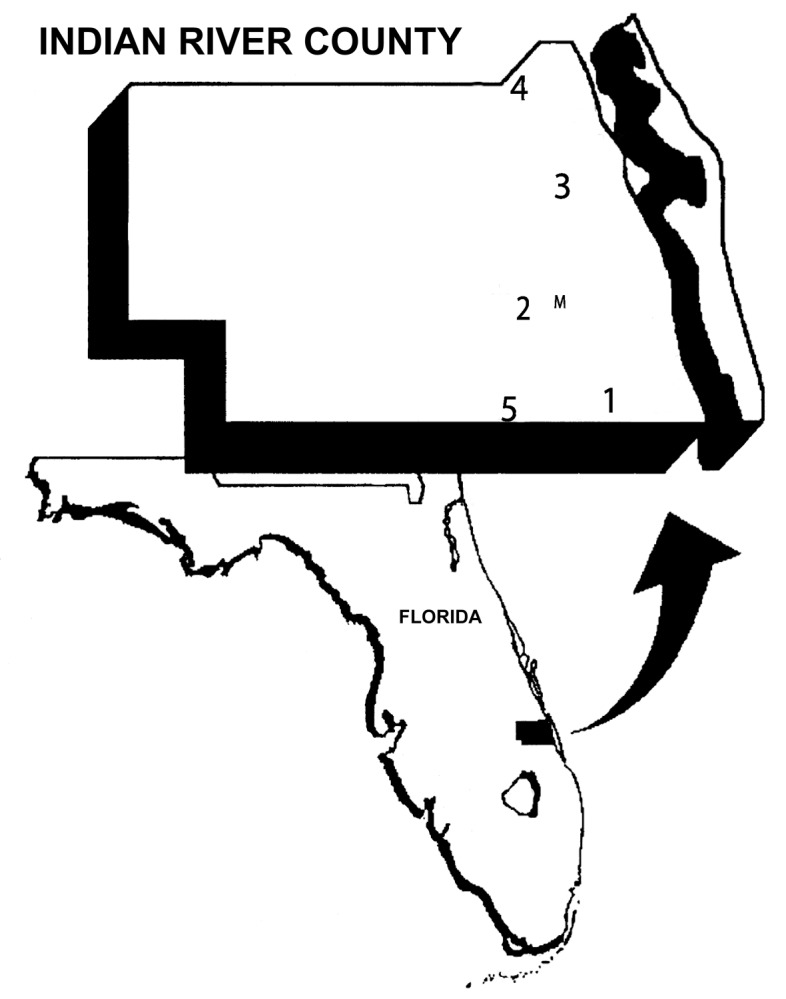
Map of Indian River County, Florida, with numbered locations of the five sentinel chicken flocks. The location of the mosquito collection site is denoted by “M.”

Sentinel chickens were used to measure SLEV transmission. The annual timing and distribution of SLEV transmission to sentinel chickens have been strongly correlated with SLEV in humans (*1*). Data derived from five sentinel flocks maintained in Indian River County were used in this study. Figure 1 is a map of the region of study and flock locations.

Sentinel chicken flocks were maintained by personnel from the Indian River Mosquito Control District. From 1986 to1990, flocks with six birds each were placed in the field by mid-June and removed at the end of December. In 1991, the year after the SLEV epidemic, surveillance was year-round. A 1.0-mL blood sample was drawn once a week from each bird during peak transmission periods (July through November) and twice a month during the rest of the year. Blood samples were assayed for hemagglutination inhibition antibodies to SLEV at the Florida Department of Health and Rehabilitative Services, Tampa Branch Laboratory. Individual chickens testing positive for hemagglutination inhibition antibodies were replaced with fresh sentinels, and the entire flock was replaced each spring.

We defined SLEV transmission intensity for each sentinel flock as the number of seropositive chickens per weekly sample. We also defined SLEV transmission incidence for each flock as a categorical data set: one, if one or more chickens were SLEV seropositive per weekly sample; or zero, if no chickens were seropositive. Data from all sentinel sites were also pooled, and SLEV transmission intensity and incidence were similarly determined.

### Mosquito Data

Western Indian River County is dominated by citrus groves intermixed with hammock “islands” of southern live oak and cabbage palm (*23*). Dense ground cover makes these hammocks an excellent daytime resting site for *Cx. nigripalpus* of both sexes and female *Cx. nigripalpus* in all gonotrophic stages (*2*). During 1986 through 1991, at least three times per week, one 20-minute collection was made approximately 2 hours after sunrise with a portable ground aspirator along a transect at a hammock site 6.4 km southwest of Vero Beach (27 38′ N, 80 27′ W, see Figure 1). Collected mosquitoes were sorted by species, categorized by sex and gonotrophic condition, and counted.

### Model Input and Validation Data

Hourly meteorologic data were assembled from National Climate Data Center archives for Vero Beach, Florida. Gaps in the record were filled with hourly data from National Climate Data Center archives for Melbourne and West Palm Beach. Solar radiation data were provided by the Northeast Regional Climate Center from analysis of the National Climate Data Center data by using the Northeast Regional Climate Center solar energy model (*24*). Topographic statistics for the Vero Beach area were generated from a 10-m cell U.S. Geological Survey National Elevation Dataset Digital Elevation Model of south-central Florida, using TarDEM version 4 routing freeware (*25*). Soil and vegetation types were derived from U.S. Department of Agriculture sources and personal inspection of the Vero Beach landscape.

The hydrology model was run from 1984 through 1995 and provided a daily series of mean WTD for the study area. Because of the channelization and water control in south Florida, the model was validated by using groundwater well measurements and surface (canal) water levels, provided by the St. John’s Water Management District. The partitioning of runoff and evapotranspiration matched bulk estimates taken from U.S. Geological Survey and St. John’s Water Management sources.

### Statistical Analysis

Univariate and bivariate logistic regression was used to associate the probability of SLEV transmission incidence with single time lags of modeled WTD and combinations of two time lags of WTD. Whole model goodness-of-fit was measured by log-likelihood ratio and the pseudo r-squared (uncertainty) coefficient. Individual parameter estimates were made by Wald’s chi-square test.

## Results

**Figure 2:**
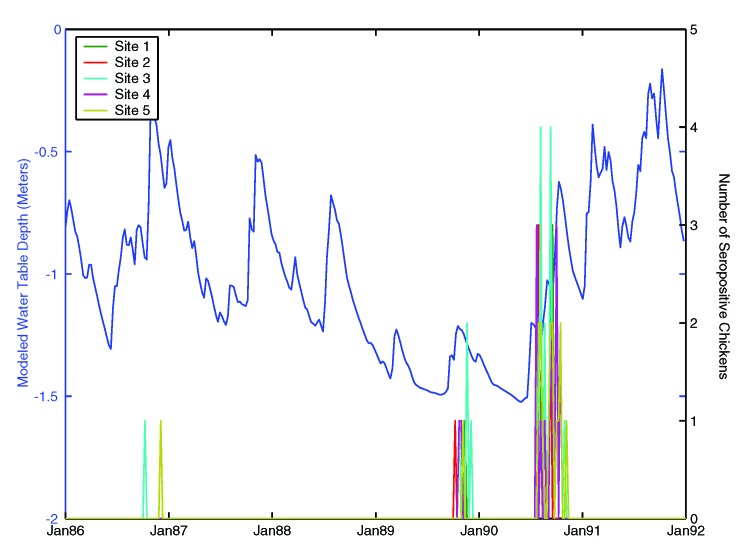
Time series of weekly seroconversion of sentinel chickens (transmission intensity) and weekly averages of modeled mean water table depth (WTD). All five sentinel flocks had *St. Louis encephalitis virus* (SLEV) transmission during the study period (1986–1991).

All five sentinel flocks had SLEV transmission recorded during the study period (1986–1991). Figure 2 provides a time series of SLEV transmission intensity and weekly averaged modeled WTD. Modeled WTD was lowest in 1989 and 1990, matching a period of drought in Vero Beach (based on Palmer Drought Severity Index records, data not shown). Three instances of SLEV transmission (the late summer and early fall of 1986, 1989, and 1990) were recorded. All three episodes occurred during a wetting period (rising of the water table) that followed a drought (low WTD). The two larger instances of SLEV transmission intensity in the Vero Beach area, 1989 and 1990, were reported during the wet conditions that followed a prolonged drought. This sequence of hydrologic conditions, antecedent and coincident with SLEV transmission, is similar to the scenario suggested by Provost (*10*).

**Figure 3:**
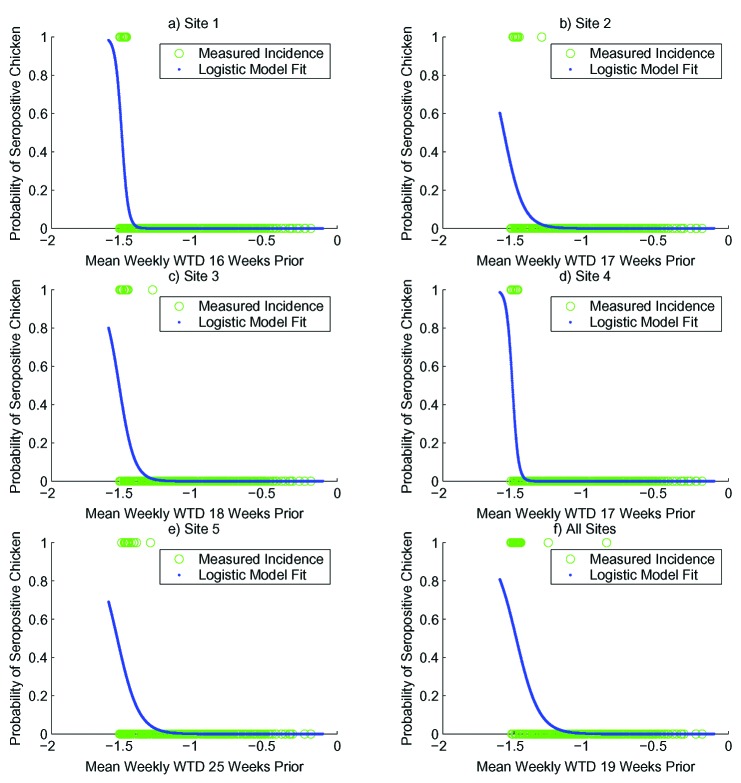
Best fit, univariate logistic regression results. a) site 1; b) site 2; c) site 3; d) site 4; e) site 5; f) all five sites, Florida.

Univariate logistic regression was performed to explore the relationship between SLEV transmission incidence and time lagged modeled WTD. A range of time lags (0–29 weeks) was tested for the individual sites and for all five sites combined. Table 1 provides a list of the best-fit logistic regression results produced by this analysis, and Figure 3 presents these results graphically. All logistic regression models were highly statistically significant (p<0.0001); in fact, a range of time lag values (generally 10–25 weeks) produced statistically significant models (p<0.001, data not shown). All five sites show the same trend: SLEV transmission incidence was strongly associated with low WTD 16 to 25 weeks before onset of SLEV transmission.

**Table 1:** Best fit results of univariate logistic regression analysis, Florida^a^

	Univariate Best Fits					
Site	Time lagged WTD	Intercept	Slope	Whole model fit p-value	Intercept	Slope
1	16	66.70	44.22	0.015	0.016	0.0001
2	17	22.99	14.63	0.0093	0.015	0.0001
3	18	28.72	18.82	0.0050	0.0069	0.0001
4	16	74.10	49.01	0.016	0.017	0.0001
5	25	20.35	13.22	0.0004	0.0010	0.0001
All five sites	19	18.55	12.49	0.0001	0.0001	0.0001

^a^WTD, water table depth; the probability of SLEV transmission incidence is represented as a function of single time lags of weekly averaged modeled WTD. Whole model goodness-of-fit was assessed by log-likelihood ratio and the pseudo r-squared (uncertainty) coefficient. Individual parameter estimates were made by Wald’s chi-square test.

A second, bivariate logistic regression analysis was performed to explore the effects of both antecedent drought and coincident wetting conditions. Modeled WTD time lags of 10 to 25 weeks were paired with modeled WTD time lags of 0, 1, 2, or 3 weeks and used together in bivariate analysis of SLEV incidence. Table 2 provides a list of the best-fit model equations resulting from this analysis. The optimal range of fits among sites is more tightly constrained when two variables are used (range 12–17 weeks before transmission for antecedent drought, 1–2 weeks before transmission for coincident wetting). The bivariate models are also more statistically significant than their univariate counterparts (based on log-likelihood ratio whole model goodness-of-fit), and the parameter estimates of both explanatory variables are statistically significant (p<0.01; p=0.068 for site 2).

**Table 2:** Best fit results of the bivariate logistic regression analysis, Florida^a^

	Bivariate Best Fits							
Site	Antecedent WTD (wks)	Coincident WTD (wks)	Whole model fit p value	Intercept	Antecedent slope	Antecedent slope p value	Coincident slope	Coincident slope p value
Site 1	12	1	0.0001	29.49	26.80	0.0005	-8.98	0.0085
Site 2	16	2	0.0001	17.83	14.13	0.0008	-3.62	0.068
Site 3	17	2	0.0001	22.11	18.64	0.0001	-5.26	0.0071
Site 4	15	1	0.0001	28.92	24.63	0.0011	-7.26	0.0096
Site 5	15	2	0.0001	21.60	23.19	0.0002	-11.37	0.0012
All five sites	17	2	0.0001	19.03	18.06	0.0001	-6.21	0.0005

^a^The probability of SLEV transmission incidence is represented as a function of two time lags of weekly averaged modeled water table depth (WTD). Whole model goodness-of-fit and individual parameter estimates were assessed as per the univariate analysis.

**Figure 4:**
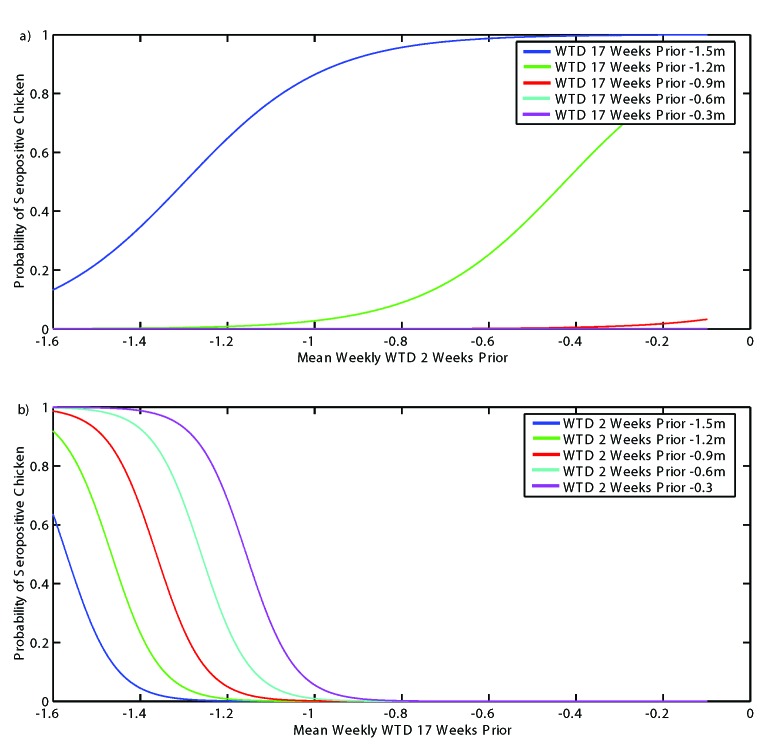
Best fit bivariate logistic regression model of *St. Louis encephalitis virus* (SLEV) incidence at all five sites combined. a) Plotted for a continuous range of modeled water table depths (WTDs) 2 weeks before transmission and fixed values of modeled WTD 17 weeks before transmission; b) plotted for a continuous range of modeled WTDs 17 weeks before transmission and fixed values of modeled WTD 2 weeks before transmission.

Figure 4 presents the bivariate model fit of SLEV incidence for all five sites combined. Figure 4a shows the logistic regression fit for a continuous range of modeled WTDs 2 weeks before transmission and fixed values of modeled WTD 17 weeks before transmission. This figure shows that antecedent drought conditions are necessary for SLEV transmission; only with a modeled WTD of <1.2 m 17 weeks before transmission is there any probability of SLEV transmission. This probability, however, is modulated by a rise in the WTD 2 weeks before transmission. This moderating effect is also shown in Figure 4b, which fixes values of modeled WTD 2 weeks before transmission but allows the conditions 17 weeks before transmission to vary. Combined, these two explanatory variables (modeled WTD 17 weeks before transmission and 2 weeks before transmission) offer a strong prediction of SLEV transmission.

The results from analysis of SLEV transmission incidence with modeled WTD were highly statistically significant but did not fully explain why the sequence of drought and wetting fosters SLEV transmission. However, a probable mechanism is suggested by mosquito collection data taken in the area.

**Figure 5:**
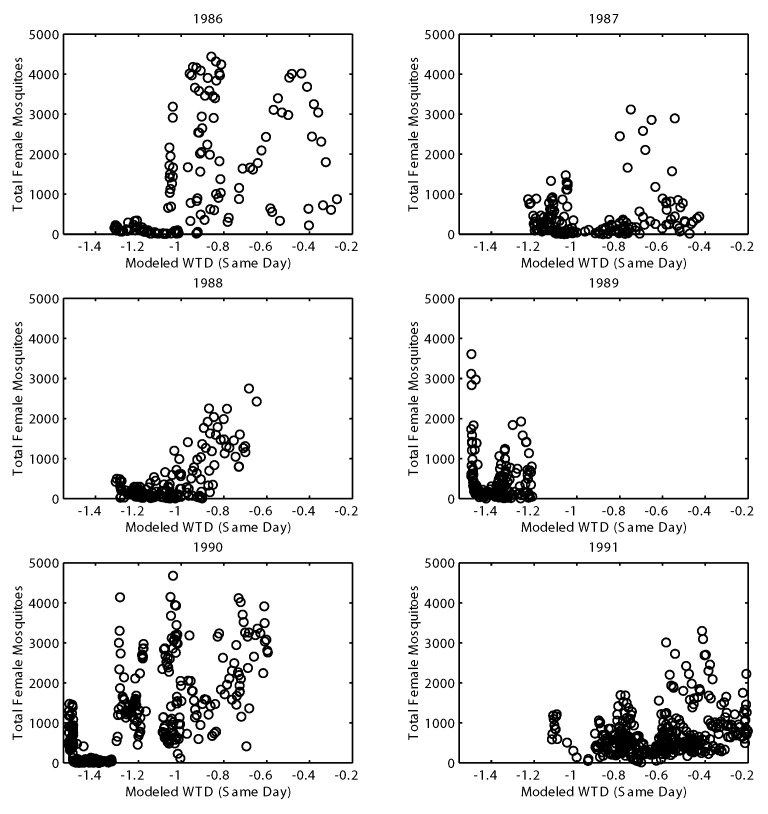
Total collected female *Culex nigripalpus* plotted as a function of modeled water table depth (WTD) (same day). Individual plots represent individual years. Mosquito collection data have been log transformed (plus one).

Figure 5 shows the distribution of total female *Cx. nigripalpus* versus mean modeled WTD for each calendar year from 1986 through 1991. Total collected female *Cx. nigripalpus* display a bimodal distribution with respect to mean modeled WTD for 3 years (1987, 1989, and 1990). Similar bimodality was evident in the yearly distributions of *Cx. nigripalpus* males and the individual female age-grades (data not shown). For 1989 and 1990, the driest years, one of the maxima of *Cx. nigripalpus* developed sharply at WTDs <1.4 m. None of the other years, including 1987, had this level of drought or this sharp bimodality. Two inferences may be drawn from these data: either the mosquito population increased at both the driest and wettest times of the year, or during the drought of 1989 and 1990, mosquitoes congregated in the hammock collection site. The latter inference is consistent with field observations that the hammocks in Indian River County and throughout south Florida provide refuge for mosquitoes during periods of drought (*23*). This “hammock” effect masks the true population dynamics; however, it illustrates an effect previously reported (*26*), namely, that drought concentrates large numbers of mosquitoes in selected refuges that also harbor large numbers of avian amplification hosts.

## Discussion

Our findings suggest the following sequence of events for SLEV transmission in Indian River County. Springtime drought restricts *Cx. nigripalpus* activity to densely vegetated hammock habitats where nestling, juvenile, and adult wild birds are found. This forced convergence of mosquito vectors and avian amplification hosts provides an ideal environment for the rapid epizootic amplification of SLEV. When the drought ends and water resources increase, infected mosquitoes and birds disperse from the hammocks, initiating the early transmission phase of the Florida SLEV cycle.

The relationship reported here between modeled WTD and SLEV incidence has only two explanatory variables and provides a simple predictive framework for forecasting SLEV transmission. To be sure, additional factors influence the dynamics of SLEV transmission. Pre-drought conditions may moderate hammock amplification by increasing or decreasing the overall abundance of mosquito vectors and avian amplification hosts. Data from other Florida counties and previous epidemics will have to be examined to elucidate how such population variability affects SLEV amplification and transmission. Future validation of the model should also include census of wild bird populations and sampling of seropositivity rates in the wild birds. Such data were not available for this study.

Whether a critical period of drought is necessary for maximum epizootic amplification also requires exploration. The 1986 data, for which the drop in WTD was short-lived and SLEV transmission was limited, suggest that the longer droughts of 1989 and 1990 were necessary for adequate amplification to produce the mosquito infection rates needed for epidemic transmission. However, if a drought persists for too long, the vectors may die, thus precluding SLEV transmission. Certainly, the biological cycles of virus, vector, and amplification hosts must be coordinated to produce an SLEV epidemic.

The mechanism of drought-induced amplification described here for Indian River County may also operate in regions outside south-central Florida that have similar epidemic SLEV transmission. In fact, the development of SLEV epidemics after drought has long been noted in many regions of the United States (*6*,*7*). Future research will attempt to quantify this relationship between drought, vector, and SLEV transmission for such regions. Differences in vector species composition, resting habitat availability, and zoonotic host prevalence will no doubt affect transmission rates and the findings of such studies.

Comparable drought-induced amplification may also occur in other arboviruses. The recent sporadic outbreak of *Eastern equine encephalitis virus* and *West Nile virus* in northern Florida, which came on the heels of a drought broken by the landfall of Hurricane Allison, suggests that these other disease systems warrant similar study.

Modeling the hydrologic cycle permits quantification of the relationship between drought and SLEV transmission and enables real-time monitoring and forecasting of SLEV transmission incidence. Using the hydrology model in conjunction with climate forecast projections, we are developing an arboviral forecast for Florida.
